# Time-Resolved Charge Detection in Transition Metal Dichalcogenide Quantum Dots

**DOI:** 10.1021/acs.nanolett.5c06351

**Published:** 2026-04-15

**Authors:** Markus Niese, Michele Masseroni, Clara Scherm, Christoph Adam, Max J. Ruckriegel, Artem O. Denisov, Jonas D. Gerber, Lara Ostertag, Jessica Richter, Kenji Watanabe, Takashi Taniguchi, Thomas Ihn, Klaus Ensslin

**Affiliations:** † Laboratory for Solid State Physics, 27219 ETH Zürich, CH-8093 Zürich, Switzerland; ‡ Research Center for Electronic and Optical Materials, 52747 National Institute for Materials Science, 1-1 Namiki, Tsukuba 305-0044, Japan; § Research Center for Materials Nanoarchitectonics, 52747 National Institute for Materials Science, 1-1 Namiki, Tsukuba 305-0044, Japan; ∥ Quantum Center, 27219 ETH Zürich, CH-8093 Zürich, Switzerland

**Keywords:** Quantum Dot, Charge Detection, Transition Metal Dichalcogenides, Tunneling, Molybdenum Disulfide

## Abstract

We investigate electronic transport through gate-defined quantum dots in molybdenum disulfide (MoS_2_) using an integrated charge detector. We observe a crossover from two weakly coupled single dots to a strongly coupled double quantum dot. In the regime of extremely weak dot-lead coupling, where the direct transport current is below the detection limit, we measure the dot occupation via charge detection and access the few-electron regime. Due to the large band gap of MoS_2_, tunneling rates can be sufficiently suppressed to resolve individual tunneling events. These results establish a platform for single-shot spin- and valley-to-charge conversion and highlight the potential of transition-metal dichalcogenide quantum dots for quantum information applications.

## Graphical abstract

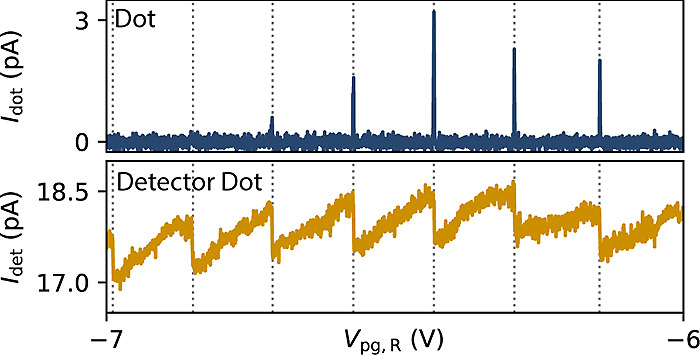

Transition metal dichalcogenides (TMDs) offer unique properties for quantum dot physics. The heavy atoms in TMDs give rise to strong spin–orbit coupling^1^ (up to 150 meV), and their large direct bandgap (1–2 eV) provides ideal conditions for creating gate-defined quantum dots (QDs). Combined with their valley degree of freedom,^2^ TMD quantum devices are a promising yet unexplored platform for applications in quantum information. Many-electron QDs based on TMDs^3^ and charge detection of incidental QDs in TMDs^4^ have been reported. However, crucial components for semiconductor qubit operation,^5^ such as single carrier QDs combined with charge detectors, have not yet been implemented. Time-resolved charge transport, which is necessary for the state readout,^6^ has not yet been realized with TMD QDs. These steps are necessary to utilize the potential of QDs in TMDs.

There have been several realizations of QDs in both MoS_2_ ^7–9^ and WSe_2_.^10,11^ Coulomb blockade was demonstrated in all of these devices, but in the many-carrier regime, where it is difficult to understand the spectrum. One of the challenges of operating QDs in TMDs in the few-electron regime is the relatively large effective mass leading to small energy level spacings.^12^ Furthermore, they have high impurity densities exceeding 10^12^–10^13^ cm^–2^.^13^ Even for advanced growth techniques where impurity densities as low as 10^10^ cm^–2^ have been reported,^14^ the mobility is still below 1000 cm^2^ V^–1^ s^–1^, which is one to 2 orders of magnitude lower than in graphene.^15^ Consequently, for bulk samples, a metal–insulator transition is observed at relatively high carrier densities of around 1.7 × 10^12^cm^–2^,^16^ where for lower densities, the charge carriers are localized. This strong localization may completely pinch off tunneling barriers and suppress the direct current through the dot long before the few carrier regime in the dot is reached.

Here, we overcome this limitation by fabricating a second dot (charge detector) capacitively coupled to the investigated dot (signal dot). This allows us to detect the charge occupation in the signal dot even when its source–drain current is below the detection limit. This technique has been pioneered in GaAs^17^ and was used in various other materials before including other systems based on van der Waals heterostructures.^18^

We detect the addition or removal of charge carriers in the signal dot by observing a shift in the resonance of the detector dot. We show that we can detect charge occupation far beyond the point where current through the signal dot becomes suppressed, and use it to detect both single dots and a double dot. The high tunability of the dots combined with the time-resolved charge detection makes this platform promising for future quantum devices.

Figure 1a shows a false-color AFM image of the device. The gates are fabricated on top of the van der Waals heterostructure. Figure 1b shows the composition of the stack, which has been fabricated using the dry-transfer method.^15^ It is made, from bottom to top, of a bottom hBN (40 nm thick), four layers of MoS_2_, obtained by mechanically cleaving a bulk crystal of natural sources (SPI supplies), a top hBN (20 nm thick), and metallic top gates made of Ti/Au (3 nm/20 nm). The stack is placed on a Si/SiO_2_ chip with a 280 nm thick oxide. The p-doped Si substrate is used as the back gate. We use graphene contacts to make an electrical connection to the MoS_2_ layer.^19^ Therefore, single layer graphene is stacked directly above the MoS_2_ flake. In Figure 1a, the two graphene flakes are sketched in gray, spanning vertically on both sides of the device and overlapping with the MoS_2_, shown in purple. The graphene and the MoS_2_ flakes overlap only in the contact area. By dry etching with CHF_3_ and O_2_, each overlapping area is divided into four individual contacts of around 5 μm^2^, thereby enabling four-terminal conductance measurements. The graphene itself is then contacted with edge contacts with Cr/Au (3 nm/70 nm),^20^ shown in red in Figure 1a. We place additional metallic gates on top of the contacts (shown in yellow in [Fig fig1]a) to accumulate carriers in the contact area independent of the carrier density in the MoS_2_, which is controlled by the silicon back gate. Using that procedure, we reach two-terminal resistances of around 2kΩ.

**Figure 1:**
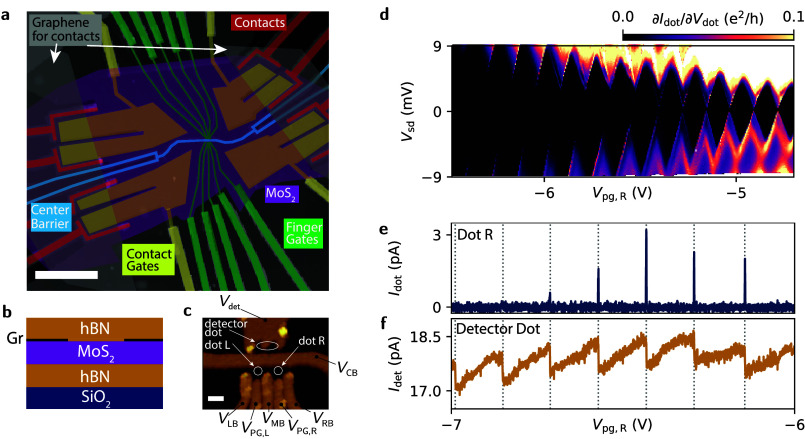
(a) False-color AFM image of the entire device. The MoS_2_ flake is purple and stretches horizontally over the whole device. The scale bar is 5 μm. (b) Cross-section of the stack with the four layers of MoS_2_ encapsulated by two flakes of hBN and partially in contact with two distinct monolayer graphene sheets forming the electrical contacts to the MoS_2_. (c) Zoom-in image of the gate structure close to the dots. The center barrier connected to V_CB_ divides the device in two halves. On the top half, the detector dot side, V_det_ controls the detector dot. On the bottom half, the signal dot side, the five gates connected to V_LB_, V_PG,L_, V_MB_, V_PG,R_, and V_RB_ control dot L and dot R. The approximate positions of dot L, dot R and the detector dot are marked. The scale bar is 100 nm. (d) Coulomb diamond measurement of the dot sitting between V_MB_, V_PG,R_ and V_RB_. (e) Current through the signal dot and (f) through the detector dot, measured simultaneously for a source drain bias *V*_sd_ = 100 μV. The steps in the detector signal always occur at the same gate voltage as the resonances of the signal dot but can even be observed when the resonances on the signal side are suppressed.

The active area of the MoS_2_ can be electronically divided into two parts by applying a negative voltage to the 50 nm wide center barrier (*V*_CB_), marked in blue in Figure 1a. To independently form dots, we tune *V*_CB_ such that the region below the central barrier is completely pinched-off. By doing so, we can independently form dots in the two parts. On both sides, we have finger gates marked in green. Figure 1c shows an AFM image of the dot area. On the lower half of the image, the signal dot side, there are five individual gates. Three of the gates act as barriers, labeled left *V*_LB_, middle *V*_MB_, and right *V*_RB_. They are located 100 nm from the center barrier. The other two gates are interleaved between the barrier gates and positioned an additional 50 nm further from the center barrier edge. They act as plunger gates *V*_PG,L_ and *V*_PG,R_. This gate geometry can be used to form either two individual decoupled dots (labeled dot L and dot R in Figure 1c) or a double dot. The upper half of Figure 1c shows the detector side. There is one large gate connected to *V*_det_ that can form a detector dot as labeled in Figure 1c.

We first focus on measurements of single dots performed in a dilution refrigerator at a base temperature of 60mK. We apply a positive voltage to the back gate to accumulate electrons in the MoS_2_ layer. By applying negative voltages to *V*_MB_ and *V*_RB_ the area below the gates gets depleted, thus forming tunneling barriers. This leads to a regime in which we observe Coulomb blockade over a wide range of plunger gate voltages *V*_PG,R_. Figure 1d shows a measurement of the differential conductance through dot R as a function of the plunger gate voltage *V*_PG,R_ and the source-drain voltage bias *V*_SD_. We observe Coulomb diamonds indicating a charging energy of around 3 meV for plunger gate voltages *V*_PG,R_ between −4.5 V and −5 V. Using a capacitor model, this corresponds to a dot radius of around 110 nm. With a typical effective mass *m*_e_^*^ ≈ 0.4–0.8 *m*_*e*_ for MoS_2_ ^12,21,22^ this corresponds to a quantization energy between 15 μeV and 30 μeV, comparable to the thermal smearing 4*k*_*B*_ *T* = 20 μeV. For more negative voltages of *V*_PG,R_, the current drops below the detection limit, even for biases up to several mV. By depleting the dot more and more, we reduce the dot–lead coupling so much that the current becomes undetectable. This behavior of the dot is consistent with previous results,^3,23^ where direct transport through the dot could only be observed at a high number of carriers in the dot. From these measurements, it is apparent that we will be unable to investigate the few-electron regime by measuring the current through the dot.

We can reach the few-electron regime by using the second side of our device for charge detection. By bringing the one big gate defining the detector region (connected to *V*_det_ in Figure 1c) close to pinch-off, a narrow channel is formed where individual conductance resonances are observed. The localized states responsible for these resonances are separated from the signal dot by the depleted area below the 50 nm wide center barrier. Thus, we can use any of the resonances of such a detector dot for capacitive charge sensing. Figure 1e shows the current *I*_dot_ through the dot R measured at low *V*_SD_ fading out for *V*_PG,R_ < −6.8 V. Whenever there is a resonance in the current *I*_dot_ through the dot, the current *I*_det_ of the charge detector, shown in Figure 1f, jumps. These jumps persist even when the direct current is completely suppressed due to opaque barriers.

In order to stay in a sensitive regime for large ranges of plunger gate voltages, we correct for the small influence of *V*_PG,R_ on the detector resonance by adjusting *V*_det_ while sweeping *V*_PG,R_. The same procedure is also performed when changing barrier gates, each with individual, slightly different, adjustment factors.

By decreasing the voltages *V*_LB_ and *V*_PG,L_ and simultaneously setting *V*_RB_ and *V*_PG,R_ to 0 V we now populate dot L. This dot is defined by the barriers *V*_LB_ and *V*_MB_ and its chemical potential can be tuned by *V*_PG,L_. The direct transport signal from dot L is completely suppressed, even for small negative voltages. We assume that this is due to some microscopic differences between dot L and R. However, we can detect the charge state with the detector. To do this, we operate the dot L similarly to dot R.

In order to experimentally establish the few carrier regime, in Figure 2a we analyze a conductivity measurement as a function of the two barrier gate voltages *V*_LB_ and *V*_MB_ at a constant plunger gate voltage *V*_PG,L_. For better visibility of the resonances, we plot the derivative of the detector current with respect to *V*_LB_. Steps in the measured *I*_det_ now appear as spikes in *∂I*_det_/*∂V*_LB_ in Figure 2a. We can shift the dot between the barriers. When making *V*_LB_ more negative, the dot moves closer to the middle barrier, thus moving the resonance to less negative voltages of *V*_MB_. This causes the diagonal lines whose slope is given by the ratio of the lever arms between the dot and the two gates. The spacing between the resonance lines increases with increasingly negative barrier gate voltages. We attribute this to the size of the dot getting smaller which leads to both a larger charging and a larger confinement energy. The last detectable resonance is marked by the red arrow.

**Figure 2:**
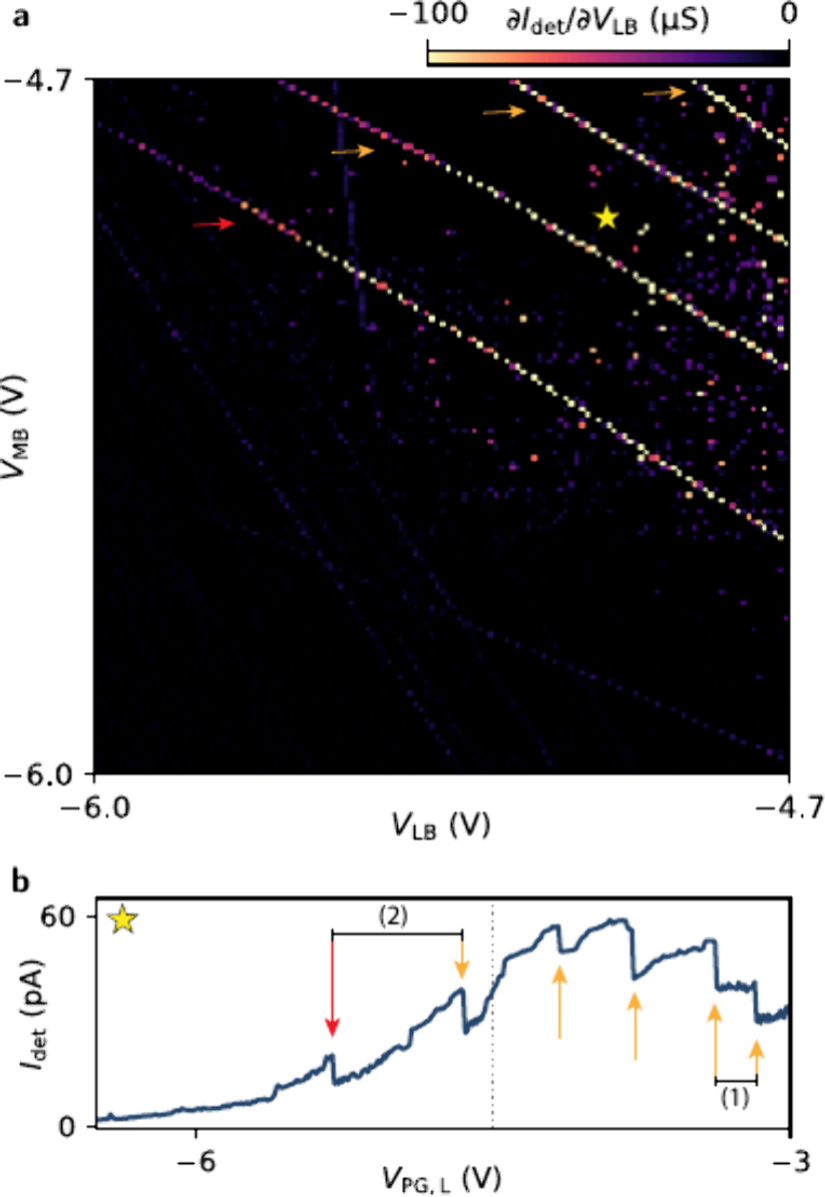
(a) Measurement of the detector current while sweeping the two barriers V_MB_ and V_LB_ and keeping V_PG,L_ constant. Orange arrows point to a set of parallel resonances, with the last one of them marked by the red arrow. The yellow star marks the barrier configuration of the measurement in (b). (b) Detector current when sweeping V_PG,L_ at fixed barrier voltages. The dotted line indicates the plunger gate voltage at which the measurement in (a) was taken.

We now continue to examine the detector signal for the same sequence of resonances by varying the plunger gate voltage at constant barrier gate voltages. Figure 2b shows this for the barrier gate voltages marked by the yellow star in Figure 2a. The dotted line in Figure 2b indicates the voltage of *V*_PG,L_ at which the map in Figure 2a was taken. In Figure 2b we observe a sequence of steps, each marked with an arrow. The separation between the charging steps increases for more negative *V*_PG,L_, ranging from 200 mV (marked by interval (1)) to 660 mV (marked by interval (2)). We note that the linear correction applied to *V*_dot_ no longer holds in this regime. This leads to the detector current level not being constant during the measurement. However, the steps can still be clearly identified.

Assuming the lever arm to be constant over the last few resonances yields an increase in charging energy from 8 meV for interval (1) to 26 meV for interval (2). The significant increase in charging energy, compared to the consistent spacing previously observed, suggests that the confinement of the dot undergoes a drastic change in this regime. This is consistent with the observation of an increasing resonance spacing, followed by the termination of the resonance sequence, as shown in Figure 2a. Such an increase in the separation between resonances is characteristic of the few-electron regime,^24^ and suggests that these features correspond to the first charge transitions of the quantum dot.

We proceed to investigate time-resolved electron-tunneling. To do so, we select a charge transition in the few-carrier regime of dot R where the tunneling rates are between 100 and 1000 Hz. Figure 3a shows the time trace of an electron tunneling in and out of the dot, leading to clearly distinguishable levels and transitions between them. By digitizing the data, we can extract the waiting times τ_in_ for tunneling into the dot and τ_out_ for tunneling out of the dot. The histogram of those waiting times is shown in Figure 3b. By fitting an exponential distribution to the histogram, we can extract the tunneling rates Γ_in_ = 1/τ_in_ and Γ_out_ = 1/τ_out_. Figure 3c shows the dependence of these rates on *V*_PG,R_ while moving through the transition from *N* to *N* – 1 charge carriers from less to more negative gate voltages. Good control over the tunneling barriers and thus achievable tunneling rates of around 200 Hz allow time-resolved measurements in our device even at zero magnetic field.

**Figure 3:**
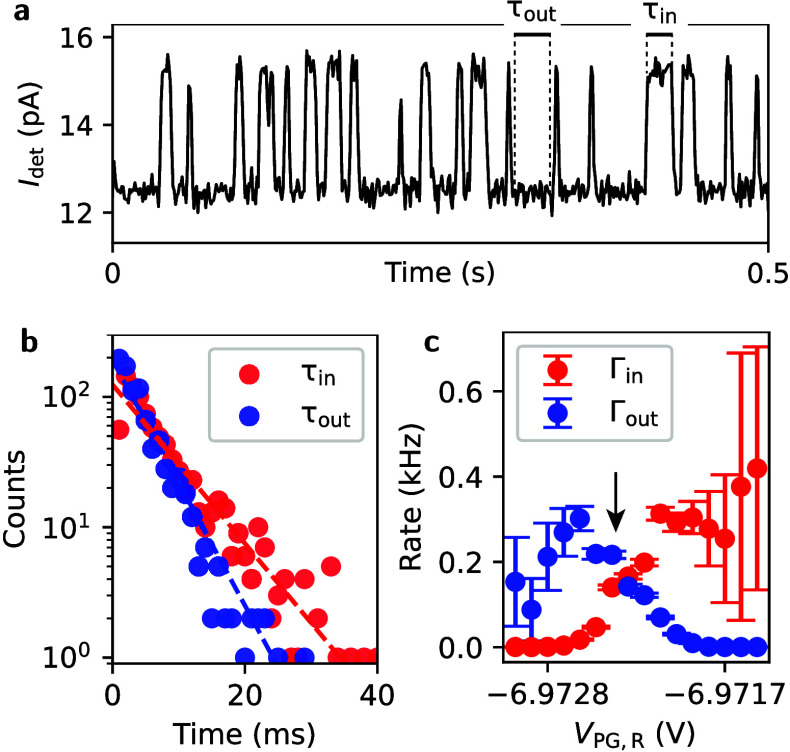
(a) Time trace of *I*_det_ showing different current levels for the electron being in and out of the dot. The lower level corresponds to the electron being in the dot. τ_in_ is the time it takes for the electron to tunnel in; τ_out_ is the time to tunnel out. (b) Histogram of tunneling in and tunneling out events at a certain *V*_PG,R_ (marked by the arrow in (c) and fits that show the exponential distribution). (c) Tunneling in (Γ_in_) and tunneling out rates (Γ_out_) as a function of the plunger gate voltage *V*_PG,R_. The arrow indicates the rates extracted from (b).

Double dots can be formed using all the barrier and plunger gates of dot R and dot L as shown in the lower half of Figure 1c. We control the chemical potentials of the two dots with *V*_*PG*,*L*_ and *V*_*PG*,*R*_, and thus independently of the barriers. With the charge detector being sensitive to both dot L and dot R, we can also use it to measure the charge state of the now formed double dot. Figure 4 shows the differential conductance through the detector as a function of both plunger gates. Similar to the single-dot data, we can observe regular dot transitions for a wide range of gate voltages for the double dot. In addition, we can see that by changing the voltage applied to the plunger gate, we can influence the interdot coupling, ranging from purely capacitive coupling in the bottom left corner to an interdot tunneling coupling of approximately *t* = 250 μeV in the top right corner. Moreover, we can control the interdot coupling independently of the potentials of the two dots using the middle barrier *V*_MB_. We present the data for the same voltages of *V*_PG,L_ and *V*_PG,R_ with *V*_MB_ = −5 V in Figure 4b and, *V*_MB_ = −4.5 V Figure 4c, respectively. The capacitive coupling strength between the dots is similar in both measurements with *e* ^2^/*C*_LR_ = 1.8 meV. The difference in tunneling coupling is clearly visible when comparing the rounded corners of the hexagons. For *V*_MB_ = −4.5 V (Figure 4c) we obtain *t* ≈ 350 μeV for the tunneling coupling, while for *V*_MB_ = −5 V (Figure 4b) *t* is too small to be extracted from the data.

**Figure 4:**
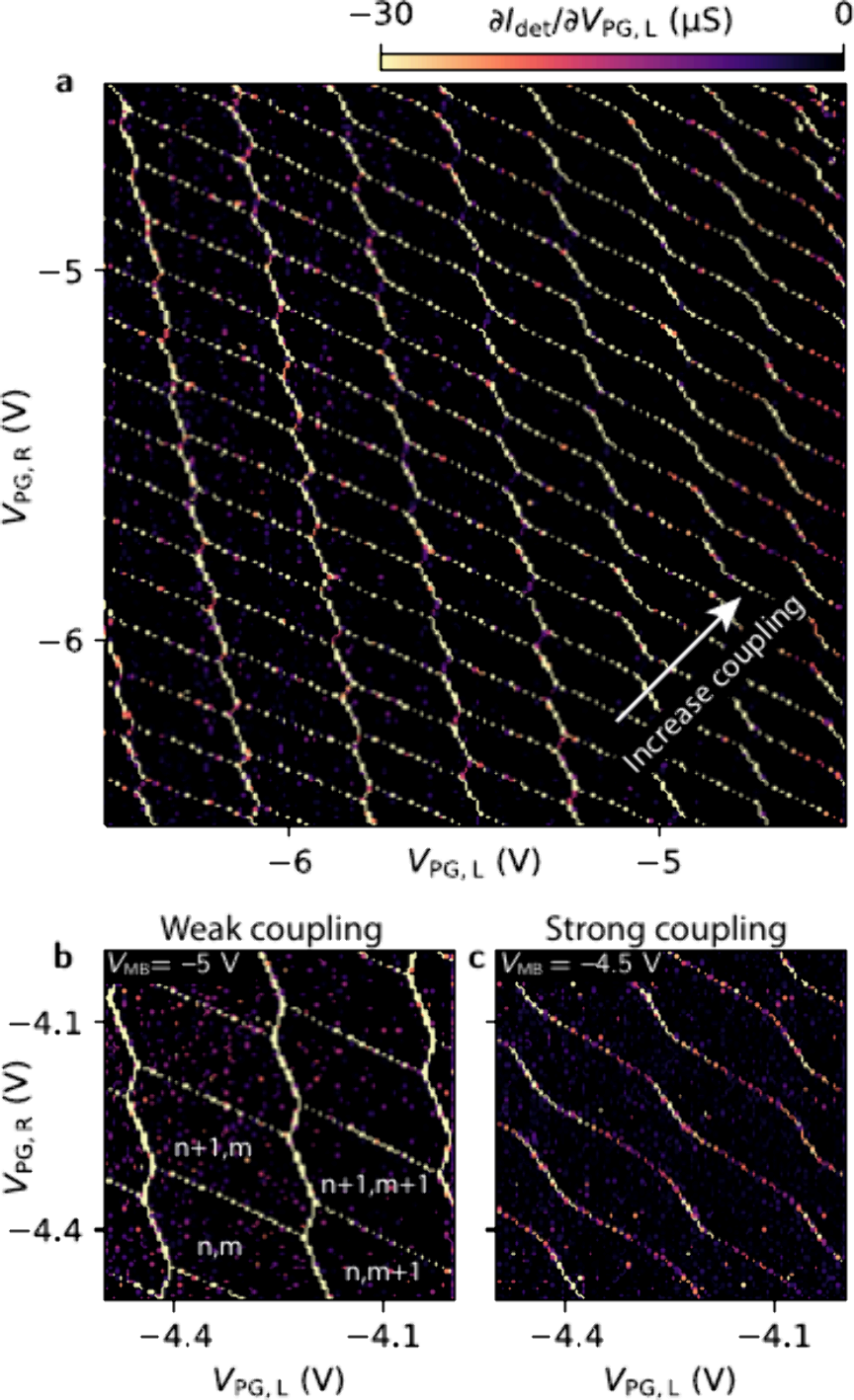
(a) Resonances of a double dot controlled by the plunger gates *V*_PG,L_ and *V*_PG,R_ with constant barrier voltages. (b) Double dot measurement with *V*_MB_ = −5 V, showing exclusively capacitively coupled dots. We indicate the number of electrons for the dot under *V*_PG,R_ by n and for *V*_PG,L_ by m. (c) The same measurement with *V*_MB_ = −4.5 V, thus increasing the tunneling coupling, rounding the resonances at their crossings. The color scale is the same for (a), (b) and (c).

In conclusion, we have presented single and double dots in MoS_2_. The dots show numerous consecutive Coulomb diamonds, demonstrating the high quality of the dot. The measurements also highlight the limitations of direct transport in this platform due to current suppression. Using a charge detector, we can discover new regimes of quantum dot operation in this material. We showed that steps in the charge detection signal correspond to resonances in direct transport. Importantly, the detection signal can be obtained even when direct transport is suppressed. The good control over the barriers and the potential allow us to do time-resolved measurements and seamlessly transform the system from a single- to a double dot regime, all while using the charge detector to measure the state. The large band gap enables strong confinement, thus enabling us to push the tunneling rates to the sub-Hz regime. These results demonstrate that charge detection in MoS_2_ is possible for single and double dots and can be optimized to perform time-resolved measurements, which is crucial to form spin qubits and investigate their lifetimes in the future.

## Data Availability

The data supporting the findings of this study, together with the code for plotting the figures, is available online through the ETH Research Collection at 10.3929/ethz-c-000795863
